# Vinyl sulfone silica: application of an open preactivated support to the study of transnitrosylation of plant proteins by S-nitrosoglutathione

**DOI:** 10.1186/1471-2229-13-61

**Published:** 2013-04-12

**Authors:** Juan C Begara-Morales, F Javier López-Jaramillo, Beatriz Sánchez-Calvo, Alfonso Carreras, Mariano Ortega-Muñoz, Francisco Santoyo-González, Francisco J Corpas, Juan B Barroso

**Affiliations:** 1 Grupo de Señalización Molecular y Sistemas Antioxidantes en Plantas, Unidad Asociada al CSIC (EEZ), Departamento de Bioquímica y Biología Molecular, Universidad de Jaén, de Jaén, Spain; 2 Instituto de Biotecnología, Universidad de Granada, Granada, Spain; 3 Departamento de Bioquímica, Biología Celular y Molecular de Plantas, Estación Experimental del Zaidín, CSIC, Granada, Spain

## Abstract

**Background:**

*S*-nitrosylaton is implicated in the regulation of numerous signaling pathways with a diversity of regulatory roles. The high lability of the S-NO bond makes the study of proteins regulated by S-nitrosylation/denitrosylation a challenging task and most studies have focused on already S-nitrosylated proteins. We hypothesize that: i) S-nitrosoglutathione (GSNO) transnitrosylation is a feasible mechanism to account for the physiological S-nitrosylation of rather electropositive sulfur atoms from proteins, ii) affinity chromatography is a suitable approach to isolate proteins that are prone to undergo S-transnitrosylation and iii) vinyl sulfone silica is a suitable chromatographic bead.

**Results:**

The combination of vinyl sulfone silica with GSNO yielded an affinity resin that withstood high ionic strength without shrinking or deforming and that it was suitable to isolate potential GSNO transnitrosylation target candidates. Fractions eluted at 1500 mM NaCl resulted in a symmetrical peak for both, protein and S-nitrosothiols, supporting the idea of transnitrosylation by GSNO as a selective process that involves strong and specific interactions with the target protein. Proteomic analysis led to the identification of 22 physiological significant enzymes that differ with the tissue analyzed, being regulatory proteins the most abundant group in hypocotyls. The identification of chloroplastidic FBPase, proteasome, GTP-binding protein, heat shock Hsp70, syntaxin, catalase I, thioredoxin peroxidase and cytochrome P450 that have already been reported as S-nitrosylated by other techniques can be considered as internal positive controls that validate our experimental approach. An additional validation was provided by the prediction of the S-nitrosylation sites in 19 of the GSNO transnitrosylation target candidates.

**Conclusions:**

Vinyl sulfone silica is an open immobilization support that can be turned *ad hoc* and in a straightforward manner into an affinity resin. Its potential in omic sciences was successfully put to test in the context of the analysis of post-translational modification by S-nitrosylation with two different tissues: mature pea leaves and embryogenic sunflower hypocotyls. The identified proteins reveal an intriguing overlap among S-nitrosylation and both tyrosine nitration and thioredoxin regulation. Chloroplastidic FBPase is a paradigm of such overlap of post-translational modifications since it is reversible modified by thioredoxin and S-nitrosylation and irreversibly by tyrosine nitration. Our results suggest a complex interrelation among different modulation mechanisms mediated by NO-derived molecules.

## Background

Proteomics has expanded to include not only protein expression profiling [1], but the analysis of post-translational modifications [2,3] and protein-protein interactions [4]. The classical experimental approach consists of a separation step based on two dimensional protein electrophoresis (2-DE) followed by an identification step that involves the cleavage with sequence specific endopeptidases and mass spectrometry (MS) for the high-throughput identification [5]. In this context affinity chromatography has been included as part of the traditional workflow either before 2-DE to selectively concentrate the sample or prior to MS for either the purification of the peptides resulting from the digestion or for the isolation of proteins bearing post-translational modifications [6].

Many studies of post-translational modifications rely on commercial resins conjugated to the proper ligand to target the particular post-translational modification being analyzed. For example beads-bound anti-phosphotyrosine antibodies have been used in phosphoproteomics [7,8] and beads coupled to lectins or saccharides in glycomics [9-12]. However this approach is constrained by the availability of the resin with the proper ligand. Supports that exploit more general affinities are also available and proteinA-IgG affinity and poly-histidine-Ni^2+^ affinity have been used for the analysis of ubiquitinated proteins, the latter implying the preparation of cells in which ubiquitin genes were replaced by a His-tag-ubiquitin coding plasmid [13-15]. A greater degree of flexibility can be achieved by exploiting the reactivity of the naturally present functional groups in biomolecules (i.e. amino, thiol and hydroxyl groups) towards classical derivatizations such as the Schiff base (reductive amination) method, N-hydroxy-succinimide method, carbonyldiimidazole method, epoxy (bisoxirane) method, ethyl dimethylaminopropyl carbodiimide method, maleimide method or hydrazide method. Commercial preactivated supports (agarose and polystyrene derivatives and, to a lesser extent, functionalized silicas) are available and there exist examples of their use [16-19], although it is not the general approach for the study of post-translational modifications.

Among the different post-translational modifications, S-nitrosylation, the addition of a nitric oxide group to cysteine thiols, is raising an increasing interest. Nitric oxide (NO) metabolism in cells has a relative short history but it has been found to be involved in a wide range of physiological and phytopatological functions. Nitric oxide exerts these functions directly or through a group of derived molecules designated as reactive nitrogen species (RNS) which are involved in post-translational modifications in cell signalling including binding to metal centres, nitration of tyrosine and S-nitrosylation of thiol groups [20]. In animals it is implicated in the regulation of numerous signaling pathways with a diversity of regulatory roles and in many cases pathophysiology correlates with dysregulation of *S*-nitrosylation [21,22]. As a classical post-translational protein modifications, S-nitrosylation is a reversible mechanism. It is not strictly dependent on enzymatic catalysis, although enzymes with nitrosylase and denitrosylase activity have been reported. In plants, evidences also support the importance of S-nitrosylation as a physiological mediator and NO is produced in most tissues [23-26]. At cellular level NO is generated and/or diffuses to cytosol, chloroplast, mitochondria, peroxisomes as well as nucleus. Nitrate reductase and L-arginine dependent nitric oxide synthase are both well recognized source of NO in plant cells [27]. Additionally NO can be produced nonenzymatically from nitrite and polyamines induce the biosynthesis of NO [26].

The high lability of the S-NO bond makes the study of proteins regulated by S-nitrosylation/denitrosylation a challenging task. Most studies have focused on already S-nitrosylated proteins and have relied on the lability of the S-NO bond to isolate them. Two different strategies are commonly assayed: i) tag derivatization and ii) resin assisted capture. The tag derivatization strategy is based on the selective derivatization of S-NO bonds with a stable tag that confers affinity for a specific resin. Thus, the biotin switch technique (BST) yields the selective biotinylation of S-nitrosylated Cys, isolation of the biotinylated proteins (i.e. S-nitrosylated proteins) by affinity chromatography on a streptavidin bound bead and their analysis by tryptic digestion and MS [28]. Variants of this technique are SNOSID (SNO-Cys Site Identification) [29] and His-tag Switch [30]. The resin-assisted capture strategy of S-nitrosylated proteins (SNO-RAC) uses the labile S-NO bond not to attach a tag and confer affinity for a resin but as a source of thiol groups that react with a thiol reactive resin [31]. Also the combination of selective fluorescent labeling of the S-NO bond and bidimensional electrophoresis has been used for the comparative study of biological samples in a variant of the DIGE technique named SNO-DIGE [32].

From a chemical point of view, S-nitrosylation reactions can be understood as the electrophilic attack of a nitro-sonium cation (NO^+^) to a thiolate [33]. In physiological conditions S-nitrosylation agents such as peroxynitrite (ONOO^-^), hyponitrite anión (NO^-^) and metal-NO complexes as well as other processes such as reaction of thiyl radicals (SO^•^) with nitric oxide radical (^•^NO) may account for the S-nitrosylation of the relatively electropositive sulfur atoms present in protein [23]. Particularly transnitrosylation (i.e. the exchange of NO^+^ from a S-nitrosylated specimen to a reactive thiolate of a target protein) seems to be an important process for in vivo protein S-nitrosylation [33]. In fact S-nitrosylated glutathione (GSNO) originated by reaction of NO with GSH is considered a natural reservoir of NO [24,34], being widely used for *in vitro* protein S-nitrosylation. Moreover, trans-nitrosylase activity has been reported for some proteins, among them hemoglobin [35] and protein disulfide isomerase [36], and particularly human thioredoxin 1 (Tdx1) that has been proposed as a "master regulator" capable of protein modulation via both redox reactions and S-nitrosylation [37]. Thus the identification of proteins that interact with transnitrosylating agents provides an additional insight complementary to that obtain by the approaches that focus on already S-nitrosylated proteins. To our knowledge such transnitrosylation studies in plants have not been published.

The availability of pre-activated materials that can be turned into an affinity support by direct reaction with the desired NO donor is a key element in the search for transnitrosylation target candidates. In this context, we have reported the biotechnological application of the reactivity of vinyl sulfone group in mild conditions toward both amino and thiol groups naturally present in biomolecules [38-40]. In the particular case of silica, the functionalization with vinyl sulfone yields a "ready to use" pre-activated material that upon reaction with both amino and thiol groups naturally present in biomolecules can be turned into an affinity support with the advantage of being obtained *ad hoc* by direct incubation with the desired macromolecule in mild conditions that preserves the biological function [40]. Thus the reaction with glutathione turned the vinyl sulfone resin into a glutathione-S-transferase (GST) affinity support that led to the one step purification of the GST and that can be used for the purification of GST tag fused proteins [40]. As an additional proof of concept, the reaction with thioredoxins (Tdx) h1 and h2 yielded two affinity chromatographic supports with different specificity in the capturing of proteins targets *in vitro:* Tdx h1 captured a transcription factor whereas Tdx h2 interacted with classical antioxidant proteins [41].

In this paper we further explore the use of the vinyl sulfone silica as an open support to address a specific proteomic problem: identification of S-nitrosylated proteins from a mature tissue (i.e. leaves from mature *Pisum sativun*) and from an embryogenic tissue (i.e. hypocotyls from *Helianthus annuus*). We hypothesize that: i) transnitrosylation by GSNO is a feasible mechanism to account for S-nitrosylation of rather electropositive sulfur atoms from proteins, ii) affinity chromatography is a good approach to isolate proteins that are prone to undergo S-transnitrosylation and iii) vinyl sulfone silica is a suitable support to be functionalized with GSNO and carry out such affinity chromatography. Our results suggest a complex interrelation among different post-translational modifications mediated by RNS.

## Results and Discussion

Studies on the formation of S-nitrosothiols in water over a pH range have revealed that the reaction occurs via the thiolate anion, not the free thiol [42], and since the typical pKa value of the Cys thiol group is between 8 and 9 transnitrosylation may be an important process for in vivo S-nitrosylation of proteins. As a matter of fact transnitrosylase activity has been reported for some proteins [35,36] and a recent study has identified 47 Tdx1 transnitrosylation target protein candidates [37]. Current approaches to study proteins regulated by S-nitrosylation/denitrosylation focus on the lability of the already present S-NO bond to isolate S-nitrosylated proteins via tag derivatization [28-30] or resin-assisted capture [31]. However in the context of transnitrosylation it is reasonable to assume that interactions play an important role that may be exploited to isolate target proteins by affinity chromatography, providing the correct election of the transnitrosylation agent and the chromatographic support. We hypothesize that i) GSNO is a feasible transnitrosylation agent candidate since it is considered a natural reservoir of NO and it is widely used *in vitro* as NO source [24,43] and ii) vinyl silica is the suitable material since it can react with GSNO to yield an *ad hoc* affinity support that withstands the high ionic strength needed to disrupt the specific interaction GSNO-target protein without swelling/shrinking [40,41]. At this point, it is important to recall that GSNO affinity chromatography on S-nitrosoglutathione-sepharose (GSNO-sepharose) has been described and that as an affinity support GSNO-vinyl sulfone silica is closely related to GSNO-sepharose since both resins are based on glutathione covalently immobilized by the amine end, being the major difference the solid support (silica vs sepharose) [44,45]. However GSNO affinity chromatography on GSNO-sepharose has been reported as an approach to screen proteins that may be modulated by NO from a different conceptual hypothesis: GSNO does not yield S-nitrosylation but S-glutathionylation and this S-glutathionylation induced by GSNO has been proposed as a general mechanism by which GSNO modifies proteins [44,45]. Both hypothesis are complementary as demonstrated by the fact that papain, creatine phosphokinase and glyceraldehyde-3-phosphate dehydrogenase are significantly both S-nitrosated and S-glutathionylated by GSNO [46], being accepted the dual role of GSNO and the relationship between S-nitrosylation and S-glutathionylation [47]. This feature adds an additional difficulty to the differential analysis. However, our experimental approach is selective for GSNO promoted S-nitrosylation since those proteins S-glutathionylated in the column (i.e. retained by the formation of a disulfide bond) are insensitive to our elution conditions (i.e. ionic strength) and remain bound to the column unless the disulfide bond is reduced, allowing the discrimination between S-nitrosylation and S-glutahionylation promoted by GSNO.

### Affinity chromatography on GSNO-vinyl sulfone silica

The S-nitrosoglutathione-vinyl sulfone silica was straightforward prepared by direct combination of glutathione with vinyl sulfone silica in a 100 mM phosphate buffer pH 7.6 (Additional file 1: Figure S1). Since vinyl sulfone is a preactivated support that reacts toward both thiol and amino groups [40], oxidized gluthione was employed and after reaction the resin was treated with β-mercaptoethanol to block the unreacted vinyl sulfone groups and with DTT to ensure the regeneration of the thiol groups that were finally S-nitrosylated by treatment with 20 mM NaNO_2_/HCl. The use of DTT may not be critical since β-mercaptoethanol is also a good reducing agent but DTT was included because it is stronger (−0.33 V vs −0.26 V) and widely used to reduce disulfide bonds in proteins. After reduction of the oxidize glutathione the content of thiol GSH was estimated by DTNB assay [48] as 1 μmol per gram of resin and the yield of the S-nitrosylation was 100% by Saville assay [49]. The storage of the GSNO-vinyl sulfone silica in cold room overnight led to a S-nitrosothiol content of 0.814 μmol g^-1^ of resin, demonstrating the stability of the *S*-nitrosoglutathione-vinyl sulfone silica.

**Figure 1:**
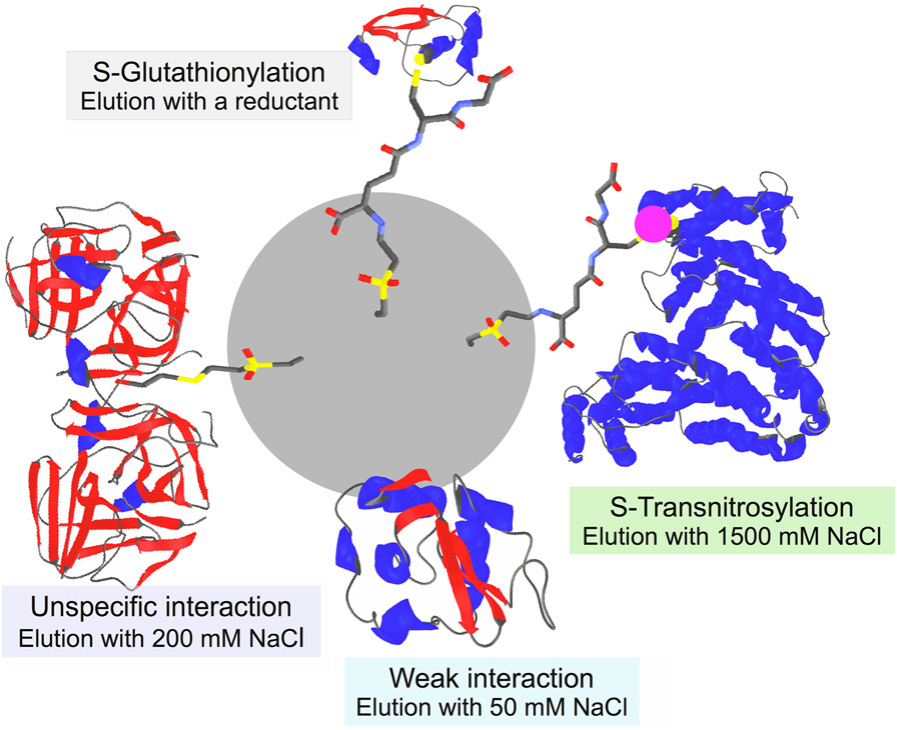
**Principle of the S-nitrosoglutathione-vinyl sulfone silica affinity chromatography.** Types of interactions between proteins and S-nitrosoglutathione-vinyl sulfone silica and conditions applied to disrupt them.

At this point it is important to recall that our experimental approach was intended to isolate and identify proteins that share a specific affinity by GSNO as a prerequisite for being target for GSNO. As depicted in Figure 1 four are the interactions responsible for the retention of the proteins in the column. Two of them are consequence of the specific interaction of GSNO with proteins to yield S-nitrosylation or S-glutathionylation, the latter by formation of a disulfide bond with the resin. Providing that S-nitrosylation can be differentiated from S-glutahionylation on the basis of the covalent nature of the latter, unspecific interactions are the main source of bias. Unlike classical affinity chromatography approaches the elution cannot be carried out with GSNO because it elutes not only protein candidates for S-transnitrosylation but also those candidates for S-glutahionylation since GSNO, as a reactive species, yields GSH, a reductant with a redox potential of −0.24 V, in the same order of that of β-mercaptoethanol, the reductant used to elute S-glutathionylated proteins from S-nitrosoglutathione-sepharose [44,45]. Bearing in mind that the use of ionic strength is a standard approach to neglect unspecific interactions and that S-transnitrosylation by GSNO implies the specific interaction between GSNO and the target protein, it is reasonable to assume that these specific interactions are stronger and require a higher ionic strength to be disrupted. Thus the discrimination of the unspecific interactions was addressed by: i) loading the sample in a buffer containing 50 mM NaCl to minimize them, ii) washing thoroughly with loading buffer to ensure the elution of the weakly bound protein and iii) including an additional step of washing with loading buffer supplemented with 200 mM NaCl to discard those proteins retained in the column by week interactions [41], even at the risk of being too strict. In fact, 200 mM NaCl may disrupt specific interactions and it has been reported to cause the dissociation of the tetrameric phosphoenol pyruvate carboxylase [50] and to yield a three-fold increase of the dissociation constant of the complex fructose-1,6-bisphosphatase:thioredoxin [51].

**Figure 2:**
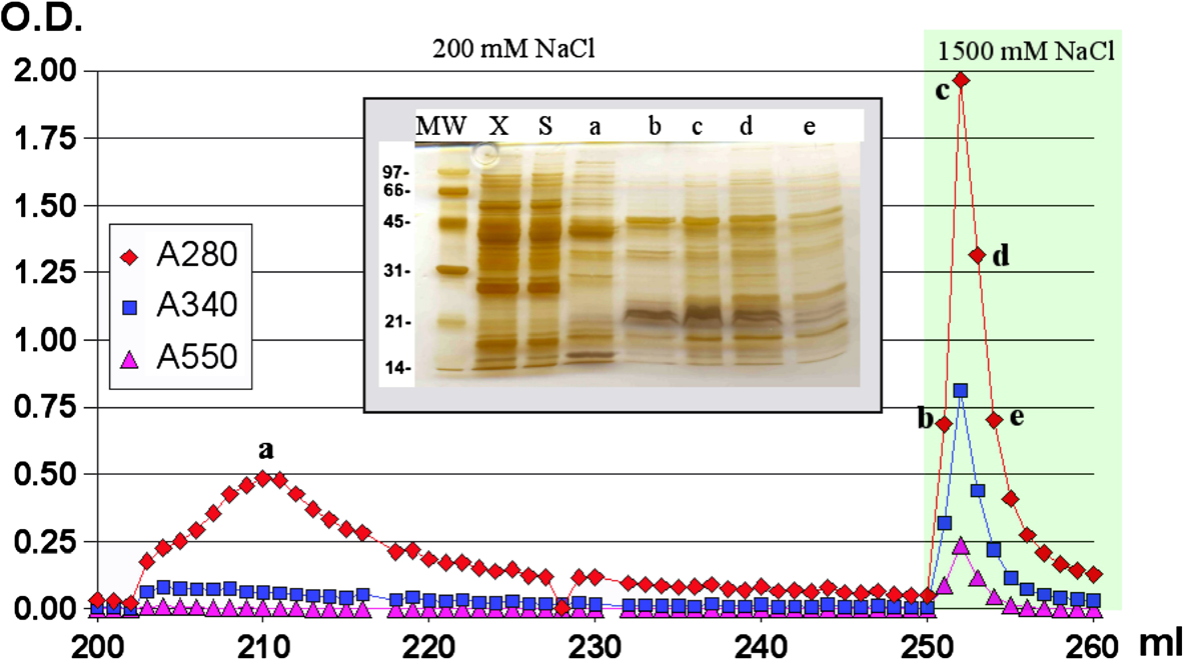
**Chromatographic fractionation.** Profile at three wavelengths (280 nm for protein and both 340 nm and 550 nm for nitrosothiol) of the pea leaf fractions eluted from the GSNO vinyl sulfone resin by 200 mM NaCl (gray) and 1500 mM NaCl (green) in 50 mM HEPES pH 7.4. Insert, SDS-PAGE of the fractions labeled as a, b, c, d and e, the crude extract (X) and the eluted fraction from the column during injection of mililiter 50 of the sample (S). The numbers on the left side of the SDS-PAGE indicate the relative molecular masses in kDa. Proteomic studies were carried out on fraction c.

S-nitrosothiols possess dual absorption maxima at 320–360 nm and ≈ 550 nm and this feature has been reported as a confirmatory evidence of S-nitrosothiol bond formation in proteins [52,53]. Hence the chromatographic process was monitored at 280 nm, 340 nm and 550 nm. Both pea leaves and sunflower hypocotyls extracts yielded a similar chromatogram (Additional file 1: Figure S2). During the loading of the sample and equilibration with loading buffer no nitrosothiol signal was detected. The increase of the ionic strength up to 200 mM NaCl yielded a broad peak of protein with no detectable S-nitrosothiol signal whereas an ionic strength of 1500 mM yielded a symmetrical sharp peak for both proteins and S-nitrosothiols as expected from a selective elution of S-nitrosylated proteins (Figure 2). SDS-PAGE revealed that the fractions comprising the peak eluted at 1500 mM NaCl shared a similar pattern and led us to focus the proteomic study on the peak fraction that showed the highest values of both protein and S-nitrosothiol signals. An important difference with other studies that use GSNO in solution is that in solution GSNO acts as a source of NO that can S-nitrosylate almost any thiol group whereas the S-nitrosylated proteins eluted from the nitrosoglutathione-vinyl sulfone silica are the result of a strong affinity by GSNO, supporting the idea that transnitrosylation by GSNO is selective and involves specific interactions with the target protein.

### Proteomic analysis

The identification was carried by the classical approach based on a separation step and an identification step by proteolysis and MALDI TOF/TOF mass spectrometry. Bidimensional separation by 2-DE of the peak fraction from pea leaves (Additional file 1: Figure S3) and sunflower hypocotyls (Additional file 1: Figure S4) led to the isolation of 33 spots and 29 spots respectively, whose tryptic digestions were analyzed by MS allowing the identification of 18 spots from pea leaves and 12 from sunflower hypocotyls. Proteins identified as GSNO transnitrosylation target candidates are summarized in Tables 1 and 2 (detailed results are tabulated as supplementary material in Additional file 1: Tables S1 and S2).

### GSNO transnitrosylation target candidates in a mature tissue

Proteins identified from the analysis of pea leaves can be grouped into four categories: i) chloroplastadic enzymes, ii) regulatory proteins, iii) proteasome subunits, and iv) others (Table 1).

**Table 1:** Summary of the positive identifications of the pea leaf proteins eluted from the GSNO vinyl sulfone resin

**Protein score C.I.%**	**Peptide count**	**Protein *species***	**MW (kDa)**	**pI**	**ID UniProtKB**	**Comments**
**Chloroplastidic enzymes**						
		**Fructose**-**1,6-bisphosphatase**			P46275	Benson-Calvin cycle enzyme.
100	9	**with transit peptide**	42.3	5.00		Regulated by thioredoxin.
100	9	**mature protein**	35.3			Reported as nitrated [88].
100	8	**nicked protein**	34.0	5.02		Reported as S-nitrosylated [43].
		*Pisum sativum*				
100	11	**Phosphoribulokinase**	39.2	5.40	P93681	Benson-Calvin cycle enzyme.
		*Pisum sativum*				
						Regulated by thioredoxin.
						Reported as nitrated [88].
		**Ferredoxin:NADP reductase**			P10933	It catalyzes the reduction of
100	9	**with transit peptide**	40.2	8.60		NADP^+^ to NADPH using the electrons provided by reduced ferredoxin.
100	8	**mature protein**	33.0	6.06		
		*Pisum sativum*				
						Reported as nitrated [89].
100	7	**Hypothetical protein**	42.8	8.19	Q9SA52	According to the UniprotKB:
		(predicted as chloroplastidic)				sugar epimerase family.
		*Arabidopsis thaliana*				Subcellular location: chloroplast.
**Proteasome**						Reported as S-nitrosylated [65].
100	6	**26S proteasome alpha-3 chain**	27.5	6.11	O24362	Involved in the structure of the proteasome particle.
		*Spinacia oleracea*				
100	8	**Proteasome subunit alpha type-7**	27.2	6.86	Q9SXU1	Involved in the structure of the proteasome particle.
		*Cicer arietinum*				
100	10	**Proteasome subunit alpha type-6**	27.5	5.83	O48551	Involved in the structure of the proteasome particle.
		*Glycine max*				
100	7	**Proteasome subunit alpha type-2-A**	25.7	5.53	O23708	Involved in the structure of the proteasome particle.
		*Arabidopsis thaliana*				
100	6	**Putative beta4 proteasome subunit**	14.6	6.40	Q93X32	Protease subunit.
		*Nicotiana tabacum*				
**Regulatory proteins**						
97.1	9	**Putative F-box protein**	38.1	9.80	Q9FVT4	F-box proteins have been associated to ubiquitination, signal transduction and regulation of the cell-cycle.
		*Arabidopsis thaliana*				
						Reported as nitrated [88].
98.8	8	**GTP**-**binding protein**	25.0	5.70	Q08147	Small GTP-binding proteins belong to a superfamily involved in the regulation as biotimers of a wide variety of cell functions.
		*Pisums sativum*,				
						Reported as S-nitrosylated in mammals [62,63].
99.7	12	**CRK1 protein.**	66.7	9.26	Q9LDC1	Cytokinine Regulated Kinase 1.
		*Beta vulgaris*				
98.8	11	**Inducer of CBF expression 1 protein**	60.8	6.51	Q1HIU3	C-repeat Binding Factor binds to CRT/DRE which actives many downstream genes that confer chilling and freezing tolerance to plants.
		*Populus suaveolens*.				
**Others**						
100	15	**Heat shock protein Hsp70**	75.8	5.19	Q1SKX2	Hsp70s are part of the machinery for protein folding and protection from stress.
		*Medicago truncatula*				
						Reported as S-nitrosylated [37].
99.7	10	**S-adenosyl-L-methionine**:	41.6	6.21	Q9LS20	It catalyzes the formation of the methyl ester of the salicylic acid, involved in the localized and systemic defense responses of plants.
		**salicylic acid carboxyl methyltransferase**				
		*Arabidopsis thaliana*				

### i) Choroplastidic enzymes

As expected from a photosynthetic tissue four chloroplastidic proteins were identified: fructose-1,6-bisphosphatase (FBPase), phosphoribulokinase (PRK), ferredoxin-NADP reductase (FRN) and an hypothetical protein that belongs to the epimerase subfamily. The identification of FBPase was expected since it has been previously reported as S-nitrosylated in *A*. *thaliana* by a different methodology: treatment of leaves with gaseous NO and biotin switch method [43]. The reliability of the identification was further validated by the fact that it led to the isolation of three isoforms: immature FBPase with its transit peptide (present in cytoplasm), mature FBPase (present in chloroplasts) and a low molecular weight form that may be the consequence of the cleavage of a highly conserved region in the amine terminus susceptible of proteolysis [54]. Similarly, FNR is isolated as the immature precursor with the transit peptide and as mature protein. From a physiological point of view both FBPase and PRK are enzymes involved in the Benson-Calvin cycle for the photosynthetic assimilation of CO_2_, both are regulated by thioredoxin and this interesting overlap between S-nitrosylation and thioredoxin modulation has been also reported by other authors [55]. Specifically the regulation of NPR1 (key regulator of defense gene expression) has been described as the result of the opposing action of GSNO and thioredoxin, the former yielding the S-nitrosylation of C151 that provokes oligomerization and inactivation of NRP1 and the latter the activation by conversion of the oligomers intro monomers [56] Additionally, the primary use of the NADPH produced by FRN is in the Benson-Calvin cycle and the NADP(H) pool or even the FNR itself have been proposed as primary signals for a feedback regulation of the photosynthetic electron transport that protect plants from an imbalance between the absorption of light energy and its use that leads to oxidative stress [57]. Recently, it has been shown that FNR is also a target of tyrosine nitration in sunflower hypocotyls under high temperature stress which provoked a reduction of its activity [58].

### ii) Regulatory proteins

Four proteins with a clear regulatory role have been identified: cytokine regulated kinase 1 (CRK1), inducer of CBF expression 1 protein (ICE), GTP-binding protein and putative F-box protein. CRK1 is a Ser/Thr kinase regulated by cytokines that has been hypothesized to be involved in hormone signaling [59]. ICE activates the expression of CBF1, that binds to the CRT/DRE DNA regulatory elements present in the promoters of the COR genes and activates their expression, being a critical element in the regulation of the genes that are expressed in cold acclimation response [60]. GTP-binding proteins are switches and timers used by plant in numerous pathways such as growth control, translational controls, vesicular transport, cytoskeletal organization and nuclear import [61]. In mammals GTP-binding protein α has been reported as nitrosylated by the biotin switch method [62,63]. F-box proteins are implicated in the ubiquitin-proteasome system and they can act as ligand receptors to modulate signaling pathways [64].

### iii) Proteasome

The proteasome is a multicatalytic protease complex consisting of the arrangement of α and β subunits to yield an α_7_β_7_β_7_α_7_ cylindrical structure. Both types of subunits have different structural and functional roles, α subunits forming the outer rings that control the entry to the catalytic chamber made of β subunits. GSNO-vinyl sulfone silica yielded the isolation of several subunits of the plant proteasome as expected from a previous study on yeast and human based on a modified biotin switch technique [65]. S-nitrosylation of the pea proteasome is reasonable given its involvement in the elimination of abnormal, misfolded or improperly assembled proteins as well as in the stress response and cell cycle control, among other important biological processes, by degradation of transcriptional regulators and cyclins [66].

### iv) Others

Other identified proteins that can not be included in the above groups but that are involved in stress response are heat shock protein Hsp70 and S-adenosyl-L-methionine: salicylic acid carboxyl methyl transferase (SAMT). The former is part of the machinery for protein folding and protection against stress [67] and it has been identified as a putative human thioredoxin transnitrosylation target [37]. The latter catalyzes the formation of the methyl ester of the salicylic acid which is engaged in plant defense and floral scent production [68].

### GSNO transnitrosylation target candidates in an embryogenic tissue

Seed germination implies the resume of metabolism and it involves the suppression of repressors, the accumulation of promoters and a new balance of hormones and tissue sensitivity to them. In this context signaling is crucial and 8 out of 11 of the GSNO transnitrosylation candidates identified from sunflower hypocotyls are involved in signaling/regulation (Table 2).

**Table 2:** Summary of the positive identifications of the sunflower hypocotyls proteins eluted from the GSNO vinyl sulfone resin

**Protein score C.I.%**	**Peptide count**	**Protein *species***	**MW (kDa)**	**pI**	**ID UniProtKB**	**Comments**
		**Ferredoxin-nitrite reductase**	67.1	6.51	P05314	Involved in nitrogen metabolism, plant regeneration and morphogenesis.
99.8	12	precursor				
100	14	fragment				Reported as nitrated [89].
		*Spinacia oleracea*				
97.6	9	**Transcriptional adapter ADA2b**	56.1	6.05	Q9ATB4	Component of transcriptional coactivator complexes. Importance in plant growth and development.
		*Arabidopsis thaliana*				
99.9	11	**Expressed protein**	43.0	5.72	Q2QWE0	Predicted.
		*Oryza sativa*				
99.9	10	**At2g18860**	34.0	5.87	Q501A1	Involved in endocytosis/exocytosis.
		**syntaxin**/**t-SNARE protein.**				
						Reported as S-nitrosylated [75].
		*Arabidopsis thaliana*				
99.9	11	**Pentatricopeptide repeat-containing protein-like protein**	42.0	8.81	Q9CAY8	RNA-binding protein. Importance in RNA editing.
		*Arabidopsis thaliana*				
99.6	10	**Hypothetical protein At2g46320**	39.3	9.37	Q8S8M6	Mitochondrial carrier family.
		*Arabidopsis thaliana*				
99.9	4	**Pectinesterase**	24.4	8.84	Q43838	It catalyzes the de-esterfication of pectin into pectate and methanol.
		*Solanum tuberosum*				
						Involved in cell elongation.
						Reported as nitrated [88].
98.7	7	**Catalase 1**	34.2	5.9	Q93XK3	H_2_O_2_ scavenger enzyme.
		(fragment)				Inhibition by NO [79].
		*Pinus pines*				
98	4	**Thioredoxin peroxidase**	30.1	8.2	Q8RVF8	H_2_O_2_ scavenger.
						Some peroxiredoxin have been reported as S-nitrosylated [80].
		*Nicotinum tabacum*				
99.7	6	**Cytochrome P450**	14.6	6.06	Q5EKR9	Biosynthetic reactions.
		(*fragment*)				NO reported to bind its Fe [87].
		*Teucrium canadense*				Reported as nitrated [89].
99.8	3	**Calmodulin***Arabidopsis thaliana*	15.6	4.20	P25069	Ca binding protein Reported as nitrated [89].

### i) *Ferredoxin nitrite reductase* (NiR)

The reliability of the assignment is based on the identification of this protein as a fragment in two different spots (Additional file 1: Table S4). NiR is a key enzyme during embryogenesis. It catalyzes the reduction of nitrite to ammonium which is then used for the synthesis of N-metabolites. Additionally, nitrite is toxic to plants and its rapid metabolism is important. In fact analyses of the expression of NiR during the regeneration process in immature embryos have revealed its importance in the process of plant regeneration [69,70].

### ii) *Transcriptional adapter ADA2b* (ADA2b)

ADA2b is a component of transcription coactivator complexes that could assist in transcriptional activation via gene-specific acetylation and it is speculated that it may be involved in DNA repair or in the generation of a DNA damage signal [71]. Its importance in plant growth and development has been reported and the hypocotyls from the ADA2b mutant are longer and the phenotype is characterized by small, dark green leaves and infertility [72].

### iii) *Syntaxin*/*t*-*SNARE protein*

SNARE (Soluble N-ethylmaleimide-sensitive fusion protein Attachment protein REceptor) proteins facilitate vesicle trafficking by forming a SNARE complex that is sufficient to overcome the dehydration forces associated with bringing two lipid bilayer together in an aqueous environment. Subsets of SNAREs occur at vesicle (v-SNARE) and target membrane (t-SNARE) and provides a mechanism of recognition, docking and fusion. However, their functions are not limited to vesicle trafficking but they are involved in signaling [73,74]. The identification of syntaxin as a GSNO transnitrosylation target is in full agreement with the work by Palmer et al. (2008) who reported that the S-nitrosylation of syntaxin is a regulator switch that controls the binding of Munc18-1 and identified Cys145 as the S-nitrosylation site by the biotin-loss technique [75].

### iv) *Pentatricopeptide repeat*-*containing protein* (*PPR*)

PPRs are RNA-binding proteins characterized by the in tandem repetition of a canonical motif comprising 35 amino acids, They are specially abundant in land plant (*Arabidopisis thaliana* genome encodes 450 PPR proteins) and plant PPR gene mutants show phenotypes that in some cases yield important defects and do not pass the embryo stage [76].

### v) *Catalase I and thioredoxin peroxidase* (*peroxiredoxin*)

Both enzymes are H_2_O_2_ scavengers and are affected by nitric oxide, suggesting a crosstalk between NO signaling and H_2_O_2_ signaling [77]. The inhibition of catalase is required for breaking the seed dormancy [78]. Nitric oxide inhibits catalase as well as several peroxiredoxins [79,80].

### vi) *Calmodulin* (CaM)

Calmodulins represent a large class of calcium sensors in plants. They have no catalytic activity but upon binding calcium they modulate a wide range of effector proteins such as protein kinases, transcriptional factors and nuclear proteins, ion transporters, channels and membrane proteins to regulate different aspects of plant stress and development [81,82]. It has been suggested that since Ca/CaM activates plant catalases it may be involved in the H_2_O_2_ homeostasis [83].

### vii) *Cytochrome P450* (CYP450)

CYP450 enzymes participate in different secondary metabolic pathways, in the biosynthesis of plant growth regulators such as jasmonic acid, gibberellins or brassinosteroids [84], being involved in hypocotyl elongation [85]. A gene encoding a CYP450 is required for auxin homeostasis and it has been suggested that it may represent a link between signaling and hormone biosynthesis [85,86]. In fact, the inhibitory effect of the the binding of NO to the heme group of CYP450 has been reported [87] and mammal CYP450 has been identified as S-nitrosylated by the biotin switch method [62].

A closer analysis of the identified proteins in the context of NO-mediated post-translation regulation reveals that in vivo tyrosine nitration under physiological conditions has been reported for FBPase, PRK, F-box proteins and pectinecterase from *Arabidosis thaliana*[88] and proteasome, FNR, cytochrome P450 and calmodulin from *Helianthus annuus* hypocotyls [89]. This interesting overlap between S-nitrosyltion and tyrosine nitration signaling was already noticed by Foster & Stamler (2004) in two proteins from liver mitochondria [90]. Furthermore, it has been proposed that peroxiredoxin II E from *Arabidopsis* possesses peroxynitrite reductase activity. However, the S-nitrosylation of this specific peroxiredoxin causes its inhibition and promotes an increase of tyrosine nitration, establishing a close connection between these two protein post-translational modifications mediated by NO-derived molecules [91]. Although S-nitrosylation is reversible and tyrosine nitration is considered as an irreversible modification, our results suggest the existence of a putative link between both post-translation modifications.

### Prediction of the S-nitrosylation site

In the context of the present work the prediction of the S-nitrosylation sites of the identified proteins may provide an additional validation of our experimental approach. It has been proposed an acid–base catalysis of S-nitrosylation with the consensus sequence (K,R,H)C(D,E) [92] but experimental results show that they are not general features and the prediction of S-nitrosylation sites remains elusive. Although protein-protein interactions play an important role that it is challenging to predict [93,94], the use of algorithms such as GSP-SNO 1.0 trained against a pool of known S-nitrosylation sites may provide some additional insight [95]. When the sequence of the proteins eluted from the GSNO-vinyl sulfone silica were analyzed the algorithm revealed the presence of S-nitrosylation sites in 19 out of 27 of the proteins/subunits identified as potential GSNO targets (Table 3). It failed to find S-nitrosylation sites for GTP-binding protein, despite it has been reported as nitrosylated in mammals [62] and for proteasome subunits alpha 2 and alpha 6, NiR, PPR, catalase 1, CYP450 and CaM. For proteasome subunit alpha 2 this result was expected since no Cys is present in its sequence and the isolation of both alpha 2 and alpha 6 can be explained on the basis that proteasome is an α_7_β_7_β_7_α_7_ complex and their interaction with subunits alpha 3 and alpha 7 respectively withstood the washing with 200 mM NaCl. The negative result for catalase and CYP450 is also reasonable since the sequences submitted to prediction are those in UniProtKB which consist of a fragment of the proteins and because the well known the interaction of NO with heme group can not be predicted by the algorithm.

**Table 3:** Prediction of the S-nitrosylation site output by GSP-SNO 1.0 for the proteins identified as GSNO transnitrosylation target candidates

**Protein species**	**ID UniProtKB**	**Position sequence**
**Fructose-1,6**-**bisphosphatase**	P46275	173
*Pisum sativum*		LGTEEQR**C**IVNVCQP
**Phosphoribulokinase**	P93681	17
*Pisum sativum*		GLAADSG**C**GKSTFMR
**Ferredoxin**-**NADP reductase**	P10933	108
*Pisum sativum*		DSKTVSL**C**VKRLVYT
**Hypotethical protein**	Q9SA52	176
*Arabidopsis thaliana*		KSDILPH**C**EEDAVDP
**Proteasome alpha**-**3 chain**	O24362	186
*Spinacia oleracea*		LKLSEMT**C**REGIIEV
		42
		GTAVGIK**C**KDGIVLG
**Proteasome alpha type-7**	Q9SXU1	74
*Cicer Arietinum*		DDHIALACAGLKADA
**Proteasome alfa type-6**	O48551	NONE
*Glycine max*		
**Proteasome alfa type-2-A**	O23708	NONE
*Arabidopsis thaliana*		
**Putative beta-4 proteasome subunit**	Q93X32	3
*Nicotina tabacum*		*****ME**C**VFGMVGN
**Putative F-box protein**	Q9FVT4	173
*Arabidopsis thaliana*		SAKILSG**C**PILEWPM
**GTP**-**binding protein**	Q08147	NONE
*Pisum sativum*		
**CRK1**	Q9LDC1	432
*Beta vulgaris*		FSTEPLA**C**DPSTLPK
**Inducer of CBF epression 1 Protein**	Q1HIU3	223
*Populus suaveolens*		ELPPALS**C**FTRLDEI
**Heat shock proteins Hsp70**	Q1SKX2	170
*Mendicago truncatula*		NGNVKLD**C**PAIGKSF
		371
		RAKFEEL**C**SDLLDRL
**S-adenosyl-L-methionine: salicylic acid**	Q9LS20	301
**carboxyl methyltransferase-like**		DDNLDQS**C**RFEVIRK
*Arabidopsis thaliana*		
**Ferredoxin-nitrite reductase**	P05314	NONE
*Spinacia oleracea*		
**Transcriptional adapter ADA2b**	Q9ATB4	356
*Arabidopsis thaliana*		KEAQVAG**C**RSTAEAE
		429
		SESEKRL**C**SEVKLVP
**Expressed protein**	Q2QWE0	8
*Oryza sativa*		MELGQPG**C**PVVQLPD
		27
		TGRLPTA**C**LGVGVKP
**At2g18860 syntaxin**/**t-SNARE protein**.	Q501A1	45
*Arabidopsis thaliana*		REKKDEI**C**KELQAAL
**Pentatricopeptide repeat-containing**	Q9CAY8	NONE
**protein-like protein**		
*Arabidopsis thaliana*		
**Hypothetical protein At2g46320**	Q8S8M6	86
*Arabidopsis thaliana*		SNSAPGM**C**RITGSAS
		284
		AVAAAAT**C**PLDVAKT
**Pectinesterase**	Q43838	162
*Solanum tuberosum*		AAVVFQK**C**QLVARKP
		196
		TGTSIQF**C**DIIASPD
**Catalase 1** (fragment)	Q93XK3	NONE
*Pinus pines*		
**Thioredoxin peroxidase**	Q8RVF8	3
*Nicotinum tabacum*		MA**C**SASSTAL
		124
		PLDFTFV**C**PTEITAF
**Cytochrome P450** (fragment)	Q5EKR9	NONE
*Teucrium canadense*		
**Calmodulin**	P25069	NONE
*Arabidopsis thaliana*		

For the chloroplastidic enzymes, the position shown is referred to the mature protein, except for the hypothetical protein.

In pea leaves a single S-nitrosylation site is predicted in 10 proteins and two sites only in proteasome alpha-3 chain and heat shock protein Hsp70. However, in sunflower hypocotyls two S-nitrosylation sites are predicted for 5 proteins. Regardless the significance of this difference, the number of putative S-nitrosylation sites per protein is in full agreement with the fact that in physiological conditions the modification of one or only few Cys thiols is responsible for the modulation. The prediction also confirms the intriguing overlap between S-nitrosylation and thioredoxin modulation of chloroplast enzymes [55]. Thus, the S-nitrosylation site predicted for chloroplastidic FBPase is C173, which is involved in the disulfide bond responsible for the light regulation of the enzyme via thioredoxin reduction [96] and for PRK is C17, equivalent to C16 in the spinach counterpart where it forms the disulfide bond with C55 involved in the regulation by thioredoxin [97].

## Conclusions

Our results support the potential of vinyl sulfone silica as an open preactivated support that can be turned *ad hoc* and straightforward into an affinity resin with a clear potential to tackle numerous biological problems ranging from protein-protein interactions to target identification or drug discovery (by immobilizing the target and assaying a mixture of potential drugs). In the context of protein S-nitrosylation, the combination of GSNO with vinyl silica yields an affinity chromatographic support suitable to isolate GSNO transnitrosylation target proteins. Only those proteins with strong affinity by GSNO withstand the stringent experimental conditions and they are eluted at high ionic strength and as S-nitrosyltated, supporting the idea that transnitrosylation by GSNO is selective and involves strong and specific interactions with the target protein. The proteomic analysis of the eluted proteins reveals that they are relevant from a physiological point of view and differ with the tissue. Thus, regulatory proteins are the most important group in an embryogenic tissue such as hypocotyls whereas in a mature photosynthetic tissue four key chloroplatic enzymes have been identified. It must be pointed out that the isolation of FBPase, GTP binding protein, proteasome, heat shock Hsp70, syntaxin, catalase I, thioredoxin peroxidase and cytochrome P450 that have already been reported as S-nitrosylated by other techniques can be considered as internal positive controls that support the consistency and validate our experimental approach. Additionally, the prediction of the S-nitrosylation site succeeded for 19 out of 26 sequences. The facts that 19 proteins have also been identified as target for tyrosine nitration and that for FBPase and PRK the putative target cysteine is part of the disulfide bond involved in the photomodulation via thioredoxin suggest a complex interrelation among different modulation mechanisms, FBPase being a peculiar case since it is modulated by thioredoxin, GSNO and tyrosine nitration.

## Methods

### Synthesis and characterization of GSNO-vinyl sulfone silica

Vinyl sulfone silica was obtained as described previously [40]. An amount of 1 g of vinyl sulfone silica was incubated at room temperature with 6 μmol of GSSG in 0.1 M phosphate buffer pH 7.6 (50 ml) for 24 h and stirring. The unreacted vinyl sulfone groups were blocked and GSSG was reduced by addition of 565 μL of ethanethiol (7 hours at room temperature) and then with 90 μmol of DTT (2 h at room temperature) to yield GSH-vinyl sulfone silica. The resin was rinsed with 10 mM HCl (1000 ml) and then incubated in 15 ml of 10 mM NaNO_2_ in 10 mM HCl (1 h in dark at room temperature) and preserved in those conditions until use.

The content of GSH bound to the resin was determined by quantification of the thiols groups by DTNB assay [48]. Briefly, GSH-vinyl sulfone silica was thoroughly washed with water, resuspended in water and then 50 μl then transferred to a cuvette containing 100 μM of DTNB in 100 mM Tris–HCl pH 8. After incubation (5 min at room temperature) the optical absorbance at 412 nm was measured and interpolated in a GSH calibration curve.

The content and stability of S-nitrosothiol groups was assayed by the Saville reaction based on the Hg assisted hydrolysis of the SNO group to yield HNO_2_ that reacts to form an azo dye from sulphanilamide and N-1-naphtylelthylendiamine [49]. Briefly, 500 μl of a suspension of nitrosylated resins were incubated with 1% (w/v) sulphanilamide and 0.2 % (w/v) HgCl_2_ in 500 mM HCl (200 μl, 5 min). The optical absorbance at 540 nm was measured after addition of 200 μl of 0.2% N-1-naphtylelthylendiamine in 500 mM HCl and interpolated in a GSNO standard curve. Samples were also measured in absence of HgCl_2_ and those values were taken as blanks.

### Plant material and growth conditions

Pea (*Pisum sativum* L., cv. Lincoln) seeds were obtained from Royal Sluis (Enkhuizen, The Netherlands). Seeds were surface sterilized with 3% (v/v) commercial bleaching solution for 3 min, then they were washed with distilled water and germinated in vermiculite for 14 d under greenhouse conditions (28–18°C, day–night temperature; 80% relative humidity). Healthy and vigorous seedlings were selected and grown in aerated optimum-nutrient solutions for 15 d [98].

Sunflower (*Helianthus annuus* L.) hypocotyls were grown as described by Chaki et al. (2009) [89]. Seeds were obtained from Koipeson Seeds SA (Sevilla, Spain), sown in wet vermiculite and grown for 9 d with 16/8 ligh/dark at 20°C.

### Preparation of crude extracts

Pea leaves or sunflowers hypocotyls were ground in liquid nitrogen using a mortar and pestle. The resulting coarse powder was transferred into 1/5 (w/v) extraction medium of 100 mM Tris–HCl buffer pH 7.5, containing 5% (w/v) sucrose, 7% (w/v) PVPP, 0.05% (v/v) Triton X-100, 0.1 mM EDTA, 15 mM DTT and 1 mM PMSF. The crude extracts were then filtered through one layer of Miracloth (Calbiochem, San Diego, CA, USA), centrifuged at 20000 g (15 min, 4°C). Protein precipitation of the resulting supernatant was carried out by incubation in 70% (v/v) acetone overnight at 4°C and stirring. The mixture was centrifuged at 16000 g (10 min, 4°C) and the pellet was resuspended in 50 mM HEPES pH 7.6 supplemented with 10 mM EDTA.

### Affinity chromatography

A volume of 150 ml of crude extract of pea leaves or sunfower hypocotyls in 50 mM HEPES pH 7.6 supplemented with 10 mM EDTA and 50 mM NaCl, the optical density at 280 nm, 340 nm and 550 nm being 4.5, 0.37 and 0.15 respectively was injected into a GSNO-vinyl sulfone silica column. After equilibration with 50 mM HEPES pH 7.6 with 50 mM NaCl to remove the non-bounded proteins, the column was washed with 200 mM NaCl in the same buffer. Elution was carried out by increasing the ionic strength of the buffer to 1500 mM NaCl and 1 ml fractions were collected. Finally, the column was cleaned with 2000 mM NaCl and 100 mM DTT.

### Proteomic analysis and prediction of the S-nitrosylation site

Proteomic analyses were carried at the Proteomic Service from the University of Córdoba as described previously [89]. Briefly, proteins were separated by two-dimensional gel electrophoresis, isoelectric focusing being carried out with precast IPG-gels pH 3–10 loaded with 100 μg of proteins. Identified spots in the gel were automatically recovered using Investigator ProPic Protein Picking Workstation (Genomic Solutions). They were digested with trypsin using an Investigator ProGest Protein Digestion Station (Genomics Solutions) and analyzed by a MALDI TOF-TOF Mass Spectrometer (Proteomics Analyzer from Applied Biosystems) in a range mass-to-charge ratio (m/z) of 800–4000 Da and in automatic mode. Internal calibration of the mass spectra was performed using the m/z of the peptides from porcine trypsin autolysis (mass MH^+^ = 842.509, mass MH^+^ = 2211.104), given a precision in the m/z ratio of 20 ppm. From each sample, the three spectrums with the highest m/z ratios were selected. The protein was identified by combining the MS spectrum with the corresponding MS/MS using the MASCOT program from the database of MatrixScience (http://www.matrixscience.com/). The following search parameters were applied, limiting the taxonomic category to green plants: a mass tolerance of 100 ppm and one incomplete cleavage were allowed; complete alkylation of cysteine by carbamidomethylation and partial oxidation of methionine.

Criteria to consider an identification as positive were: i) agreement between both molecular weight and isolectric point inferred from the 2D gel and those reported in literature or predicted from the primary structure and ii) either fragmentation or mass fingerprint yielded a protein score C.I. better than 97% (instead of the less stringent 95% cutoff generally accepted).

Prediction of the nitrysolation sites was carried out by GSP-SNO 1.0 [95]. The algorithm is not based on a simple motif approach but on a scoring strategy using an amino acid substitution matrix (matrix mutation).

## Abbreviations

ADA2b: Transcriptional adapter ADA2b; CaM: Calmodulin; CRK1: Cytokine regulated kinase 1; CYT450: cytochorme P450; 2-DE: two dimensional protein electrophoresis; DTNB: 5-(3-Carboxy-4-nitrophenyl)disulfanyl-2-nitrobenzoic acid; DTT: dithiothreitol; FRN: Ferredoxin-NADP reductase; FBPase: Fructose-1,6-bisphosphatase; GSNO: S-nitrosglutathione; GST: glutathione-S-transferase; ICE: Inducer of CBF expression 1 protein; NiR: Ferredoxin nitrite reductase; NO: nitric oxide, PPR, Pentatricopeptide repeat-containing protein; PRK: Phosphoribulokinase; RNS: reactive nitrogen species; SNARE: soluble N-ethylmaleimide-sensitive fusion protein attachment protein receptor; SAMT: S-adenosyl-L-methionine: salicylic acid carboxyl methyl transferase; Tdx: thioredoxin.

## Authors’ contributions

JCBM, BSC and AC carried out the growth of plant material, the preparation of the crude extracts, the chromatographic fractionation and the characterization of the GSNO vinyl sulfone silica. FJLJ, MOM and FSG designed and synthesized the GSNO vinyl sulfone silica and its use as an affinity chromatography support. FJC and JBB conceived the study and oversaw the proteomic analysis. FJLJ was responsible for the proteomic analysis and drafted the manuscript. All authors read and approved the final manuscript.

## Supplementary Material

Additional file 1: Figure S1 Synthesis of the S-nitrosoglutathione-vinyl sulfone silica. **Figure S2.** Profile of the affinity chromatography of the pea leaves crude extract monitored at three wavelengths to detect both protein (A280) and S-nitrosothiol (A340 and A550) signal and the types of interactions that are disrupted. **Figure S3.** Bidimensional electrophoresis of the peak fraction of pea leaves eluted. **Figure S4.** Bidimensional electrophoresis of the peak fraction of sunflower hypocotyls. **Table S1.** Summary of the positive identifications by mass spectrometry of the peak fraction of the pea leave sample eluted from a GSNO-vinyl sulfone resin that showed the highest protein and Snitrosylation signals. **Table S2.** Summary of the positive identifications by mass spectrometry of the peak fraction of the sunflower hypocotyls sample eluted from a GSNO-vinyl sulfone resin that showed the highest protein and S-nitrosylation signals. Click here for file
