# Supplementary material for: Vinyl sulfone silica: application of an open preactivated support to the study of transnitrosylation of plant proteins by S-nitrosoglutathione
Source: BMC Plant Biol. 2013 Apr 12;13:61. doi: 10.1186/1471-2229-13-61 (PMC3639107; doi:10.1186/1471-2229-13-61)
Supplement: Additional file 1: Figure S1 — Synthesis of the S-nitrosoglutathione-vinyl sulfone silica. Figure S2. Profile of the affinity chromatography of the pea leaves crude extract monitored at three wavelengths to detect both protein (A280) and S-nitrosothiol (A340 and A550) signal and the types of interactions that are disrupted. Figure S3. Bidimensional electrophoresis of the peak fraction of pea leaves eluted. Figure S4. Bidimensional electrophoresis of the peak fraction of sunflower hypocotyls. Table S1. Summary of the positive identifications by mass spectrometry of the peak fraction of the pea leave sample eluted from a GSNO-vinyl sulfone resin that showed the highest protein and Snitrosylation signals. Table S2. Summary of the positive identifications by mass spectrometry of the peak fraction of the sunflower hypocotyls sample eluted from a GSNO-vinyl sulfone resin that showed the highest protein and S-nitrosylation signals. [file 1471-2229-13-61-S1.pdf]

Figure S1. Synthesis of the S-nitrosoglutathione-vinyl sulfone silica.- (i) reaction of vinyl silica with oxidized glutathione (GSSG) in 100 mM phosphate buffer pH 7.6 at rt; (ii-iii) blockage of the unreacted vinyl sulfone groups (VS) with β-mercaptoethanol (bETSH), generation of glutathione (GSH) and further reduction with DTT; (iv) S-nitrosylation by treatment with NO_2Na/HCl to yield S-nitrosoglutathione-vinyl sulfone silica

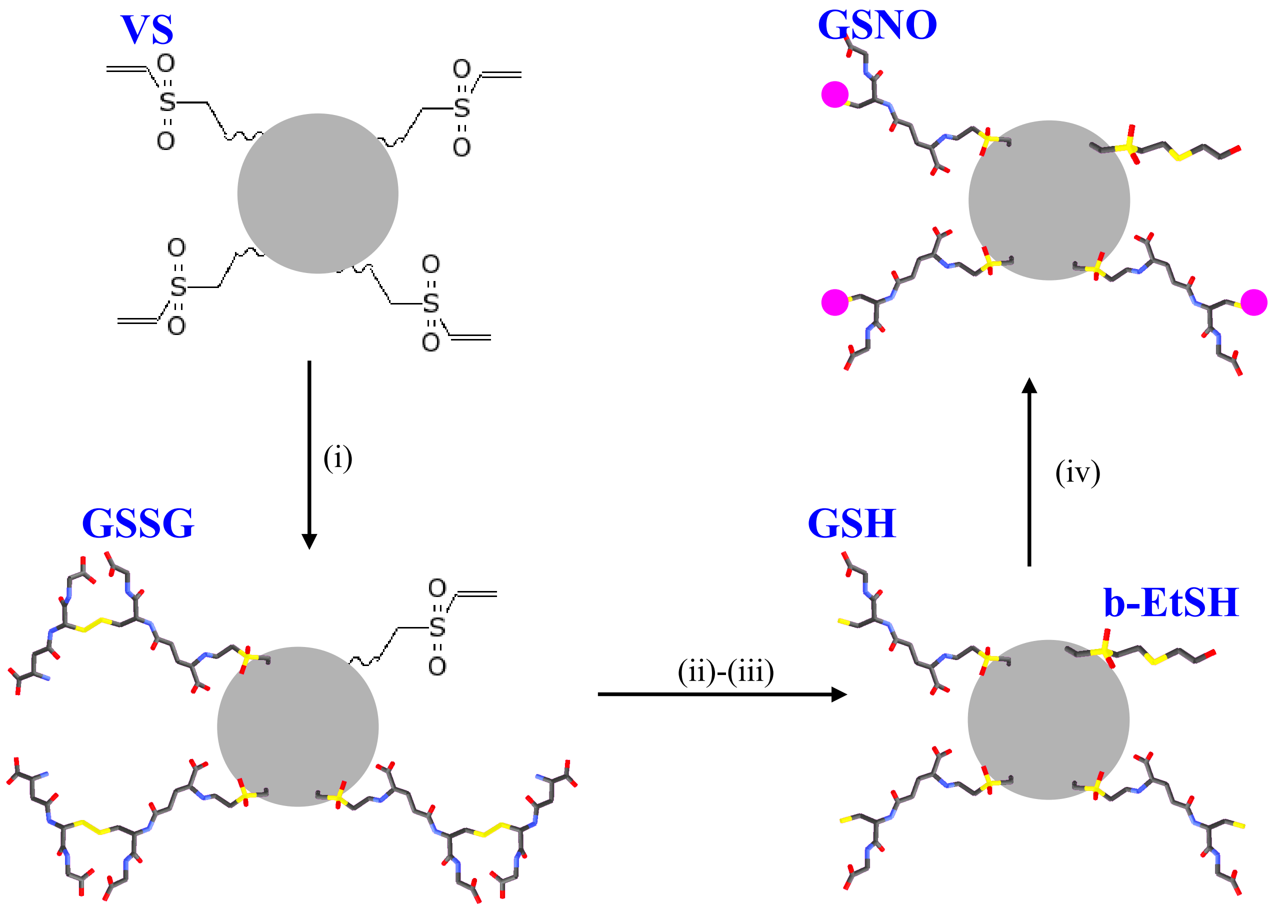

Figure S2. Profile of the affinity chromatography of the pea leaves crude extract monitored at three wavelengths to detect both protein (A280) and S-nitrosothiol (A340 and A550) signal and the types of interactions that are disrupted (insert).- A volume of 150 ml of crude extract of pea leaves in 50 mM HEPES pH 7.6 supplemented with 10 mM EDTA and 50 mM NaCl. After equilibration with 50 mM HEPES pH 7.6 with 50 mM NaCl to remove the non-bounded or weakly bounded proteins, the column was washed with 200 mM NaCl in the same buffer to discard those proteins retained by unspecific interactions and then eluted by increasing the ionic strength of the buffer to 1500 mM NaCl. Finally, the column was cleaned with 2000 mM NaCl and 100 mM DTT to removed those proteins that formed a disulfide bond with the resin.

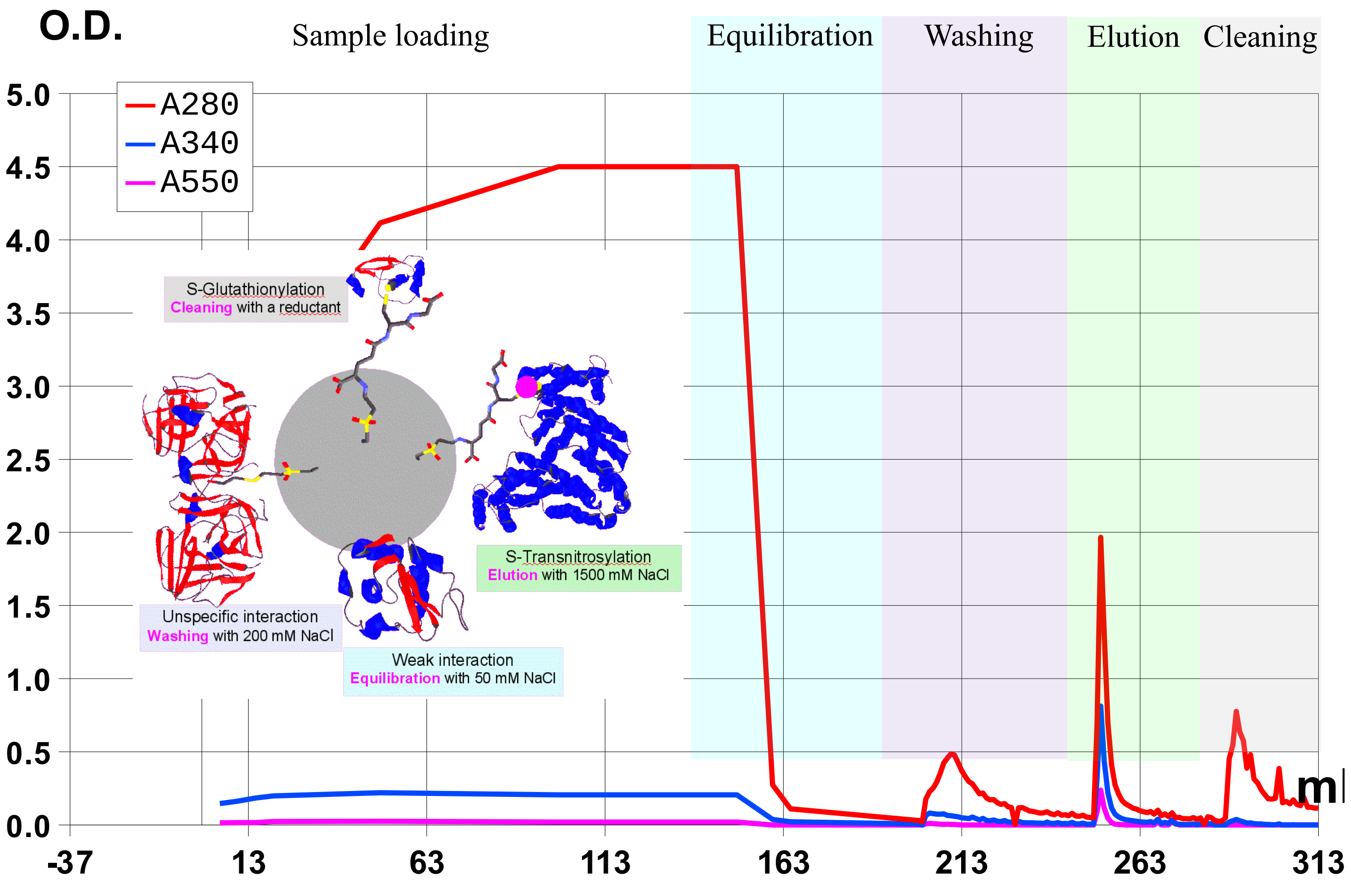

Figure S3. Bidimensional electrophoresis of the peak fraction of pea leaves eluted at 1500 mM NaCl that showed the highest protein and S-nitrosothiol signal. The gel was stained with Spypro Ruby. Molecular-mass standards are indicated on the left in kDa. Spots picked, digested with trysin and analyzed by mass spectrometry are numbered.

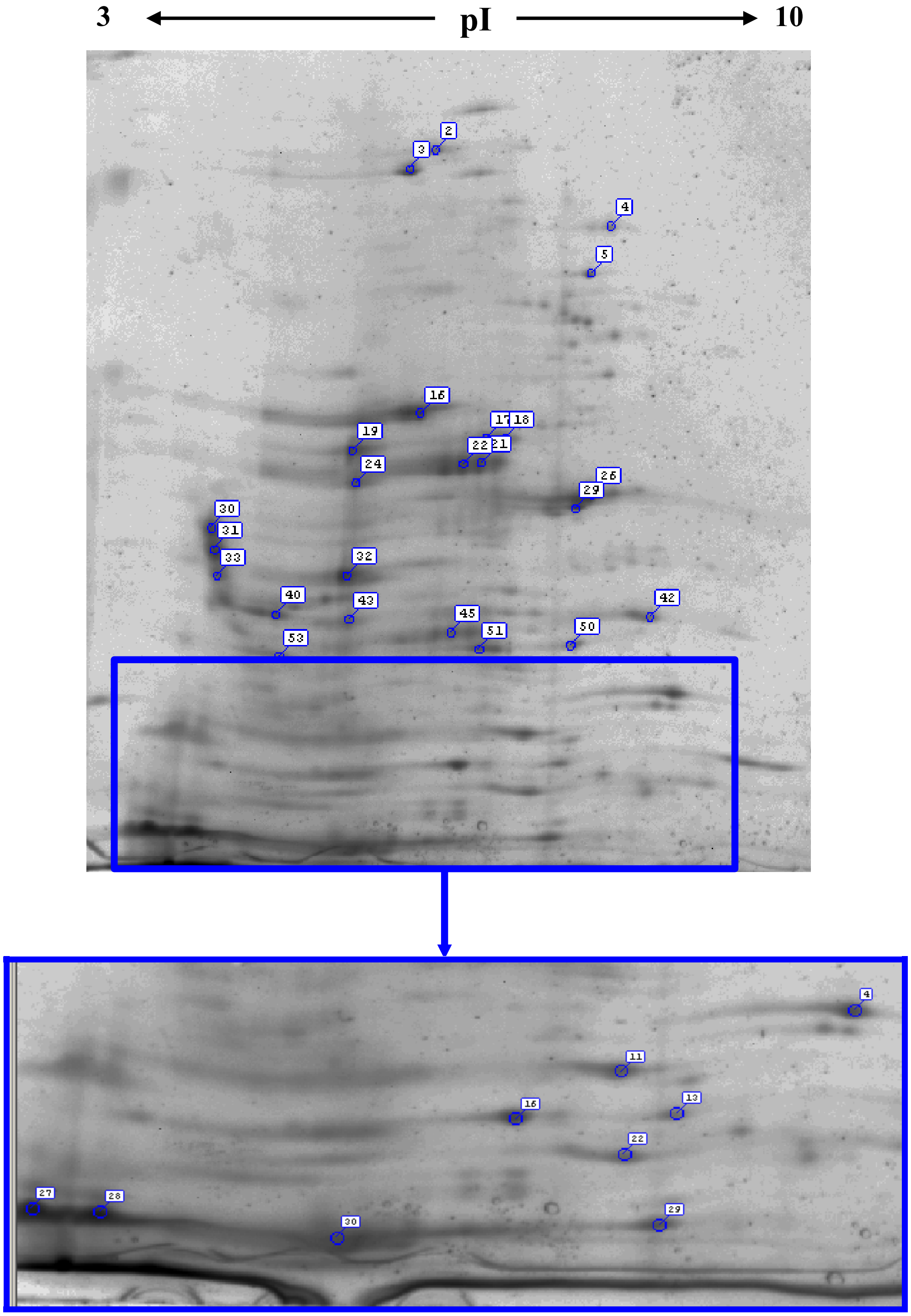

Figure S4. Bidimensional electrophoresis of the peak fraction of sunflower hypocotyls eluted at 1500 mM NaCl. (a) The gel was stained with Spypro Ruby. Molecular-mass standards are indicated on the left in kDa. (b) Spots that were picked, digested with trysin and analyzed by mass spectrometry.

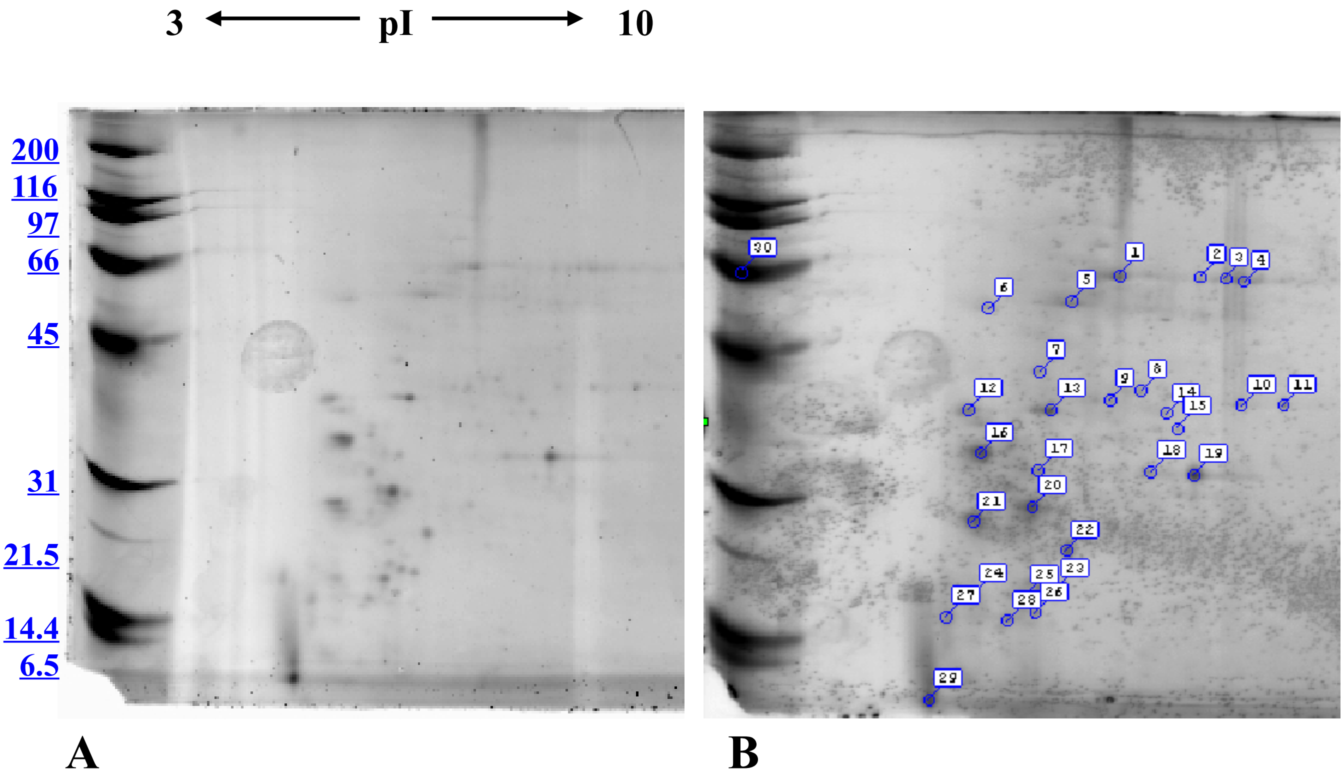

Table S1. Summary of the positive identifications by mass spectrometry of the peak fraction of the pea leave sample eluted from a GSNO-vinyl sulfone resin that showed the highest protein and S-nitrosylation signals. Criteria to consider an identification as positive were: i) agreement between both molecular weigh and isoelectric point inferred from the 2D gel and those reported in literature or predicted from the primary structure and ii) either fragmentation or mass fingerprint yielded a protein score C.I. better than 97% (instead of the less stringent 95% cutoff generally accepted). Peptides labeled as (*) were confirmed by MS/MS

**spot**	**Protein score C.I.%**	**Peptide count**	**Protein**<br>*species*	**MW (kDa)**	**pI**	**ID**	**identification method**<br>matched peptides<br>[(*) Peptides identified by MS/MS]
**5**	7	12	**CRK1 protein**.<br>*Beta vulgaris subsp vulgaris*	66.7	9.26	Q9LDC1	**Peptide mass fingerprinting**<br>KHPMTSR<br>DISSSRAR<br>NVVEGNEPK<br>RIITHANAK<br>IITHANAKSK<br>YPPSKEMDAK<br>FDNLEPESVK<br>LCGSPSDEYWK<br>MGCVFGKESLAPEGR<br>VDLPVADVVSGWDTGK<br>GVGQHWSGPLVDPASAGGPR<br>DFPPSALSLIDSLLAIDPAER
**16**	100	15	**Heat shock protein Hsp70**<br>*Medicago truncatula*	75.8	5.19	Q1SKX2	**MS/MS (total ion C.I.%: 99.998%)**<br>IAGLEVLR<br>TPVENSLR<br>IPAVQELVK<br>DNKSLGSFR<br>DIDEVILVGGSTR<br>SFAAEEISAQVLR<br>SFAAEEISAQVLRK<br>AVVTVPAYFNDSQR (*)<br>QAVVNPENTFFSVK<br>SLGSFRLDGIPPAPR<br>AKFEELCSDLLDR<br>MSEVDEESKQVSYR<br>QAVVNPENTFFSVKR<br>IINEPTAASLAYGFER<br>LSIKDIDEVILVGGSTR (*)
**17**	98.8	11	**Inducer of CBF expression 1 protein**<br>*Populus suaveolens.*	60.8	6.51	Q1HIU3	**Peptide mass fingerprinting**<br>IMHLGIK<br>NLMAQWR<br>AVNIHMFCGR [oxidation M6]<br>AVNIHMFCGRK<br>GLPAKNLMAQWR<br>MQLNDRLYTMR<br>LDEISVNPLFSSR<br>LLSPLETYPIHGR<br>GDQDSIKDLTFSPR<br>IMDQLCPSSLPSPNGQPAR

spot	Protein score C.I.%	Peptide count	Protein<br>*species*	MW (kDa)	pI	ID	identification method<br>matched peptides<br>[(*) Peptides identified by MS/MS]
							LLSPLETYPIHGRSATLFQR
**18**	99.7	10	**S-adenosyl-L-methionine:salicylic acid carboxyl methyltransferase**<br>*Arabidopsis thaliana*	41.6	6.21	Q9LS20	**Peptide mass fingerprinting**<br>LVPAFHK<br>MALTFVGR<br>NDFSLFLR<br>MALTFVGRK<br>CFISGVPGSFYSR<br>LFERYTYHLAER<br>RLASDAKPVVVETVK<br>NKPTVQFFVSLTRK<br>SITEPMLGAHFGDAIMDR<br>ASIIRSITEPMLGAHFGDAIMR
**19**	100	9	**Chloroplastic Fructose-bisphosphatase**<br>(with transit peptide)<br>*Pisum sativum*	42,3	5.00	P46275	**MS/MS (total ion C.I.%: 99.998%)**<br>HVNGSGVR<br>IYSFNEGNYK<br>YIGSLVGDFHR<br>TLLYGGIYGYPR<br>VLDIQPTEIHQR (*)<br>CVFDAKSVLSSSR<br>KLDVISNEVFSNCLR<br>VPLYIGSTEEVEKVEK<br>YIDDLKEPGPSGKPYSAR (*)
**21**	100	11	**Phosphoribulokinase**<br>*Pisum sativum*	39.2	5.40	P93681	**MS/MS (total ion C.I.%: 100)**<br>LIQKAGVK<br>GHSLESIK<br>FFYGPETYK (*)<br>DLFEQIVASR<br>FYGEVTQQMLK (*)<br>RLTSVFGGAAEPPK<br>IRDLFEQIVASR<br>KPDFEAYIDPQK<br>ANDFDLMYEQVK (*)<br>ILVIEGLHPMYDSR<br>HADFPGSNNGTGLFQTIVGLK
**24**	100	9	**Chloroplastic Fructose-bisphosphatase**<br>(mature protein)<br>*Pisum sativum*	35.3	5.02	P46275	**MS/MS (total ion C.I.%: 100)**<br>QIASLVQR<br>ANISNLTEDQK<br>IYSFNEGNYK<br>EPGPSGKPYSAR<br>YIGSLVGDFHR (*)<br>TLLYGGIYGYPR (*)<br>VLDIQPTEIHQR<br>VPLYIGSTEEVEKVEK<br>YIDDLKEPGPSGKPYSAR (*)
**26**	100	7	**Hypothetical protein**<br>(predicted as chloroplastidic)<br>*Arabidopsis thaliana*	42.8	8.19	Q9SA52	**MS/MS (total ion C.I.%: 100)**<br>ILIMGGTR<br>KILIMGGTR<br>EGHQVTLFTR<br>DQHFFASVEK<br>GALYVSASSEKK

spot	Protein score C.I.%	Peptide count	Protein *species*	MW (kDa)	pI	ID	identification method matched peptides [(*) Peptides identified by MS/MS]
							AGGFPEPEIVHYNPK<br>SSLSAEGFDVVYDINGR (*)
**29**	97.1	9	**Putative F-box protein**<br>*Arabidopsis thaliana*	38.1	9.80	Q9FVT4	**Peptide mass fingerprinting**<br>ADNSATRVR<br>WTEFAMSR<br>VKALTLEMK<br>LESGSMLELK<br>MADNSATRVR<br>GVPFPMFKVK<br>NVENMSLDVR<br>MMNFHLKINK<br>SLSHRLDLHDVIPR
**42**	100	9	**Ferredoxin:nadp+ reductase**<br>(with transit peptide)<br>*Pisum sativum*	40.2	8.60	P10933	**MS/MS (total ion C.I.%: 100)**<br>MYIQTR<br>FKPKEPYVGR<br>GIDDIMVSLAAK<br>LVYTNDAGEVVK (*)<br>RLVYTNDAGEVVK<br>KDNTFVYMCGLK<br>APENFRLDFAVSR<br>LYSIASSAIGDFGDSK<br>DPNATVIMLGTGTGIAPFR
**43**	100	8	**Chloroplastic Fructose-bisphosphatase**<br>(*nicked* form)<br>*Pisums sativum,*	34.0		P46275	**MS/MS (total ion C.I.%: 100)**<br>SVLSSSRR<br>IYSFNEGNYK<br>EPGPSGKPYSAR<br>YIGSLVGDFHR (*)<br>TLLYGGIYGYPR<br>VLDIQPTEIHQR<br>VPLYIGSTEEVEK<br>YIDDLKEPGPSGKPYSAR (*)
**45**	98.8	8	**GTP-binding protein**<br>*Pisums sativum,*	25.0	5.70	Q08147	**Peptide mass fingerprinting**<br>NIVIILVGNK<br>STIGVEFQTR<br>ANSGGYGDANQR<br>FSRNEFSLDSK<br>MANSGGYGDANQR<br>DVPTEDAKEFAEK<br>GAVGAMLVYDITKR<br>SDLENQRDVPTEDAK
**50**	100	8	**ferredoxin: nadp+reductase**<br>(mature protein)<br>*Pisum sativum*	33.0	6.06	P10933	**MS/MS (total ion C.I.%: 100)**<br>DGIDWLEFK<br>GIDDIMVSLAAK<br>LVYTNDGGEIVK<br>ITGPVGKEMLMPK<br>RLVYTNDGGEIVK<br>LGFSVSREQTNEK<br>QEEGIVVNKFKPK<br>LYSIASSAIGDFGDSK (*)

spot	Protein score C.I.%	Peptide count	**Protein** *species*	MW (kDa)	pI	ID	**identification method** matched peptides [(*) Peptides identified by MS/MS]
**51**	100	6	**26S proteasome alpha-3 chain** *Spinacia oleracea*	27.5	6.11	O24362	**MS/MS (total ion C.I.%: 99.999)** YFGAAIGK MMLPGSNR VFQIEYAAK (*) MMLPGSNRR CKDGIVLGVEK HSGMAVAGLAADGR
**Bottom part of the gel**							
**4**	100	8	**Proteasome subunit alpha type-7** *Cicer arietinum*	27.2	6.86	Q9SXU1	**MS/MS (total ion C.I.%: 99.871%)** KGNAAVGVR YIAGLQQK (*) NIEVAVMTK ALLEVVESGGK GTDNVVLGVEK GTDNVVLGVEKK NSNSIREFLEK NFKETSGQETVK
**11**	100	10	**Proteasome subunit alpha type-6** *Glycine max*	27.5	5.83	O48551	**MS/MS (total ion C.I.%: 100)** TLVQQAR NEAAEFR AAGITSIGVR LFQVEYAFK (*) HITIFSPEGR DQEAINFLEK YLGLLATGMTADAR ATSAGLKDQEAINFLEK (*) VLTTDEIDEHLTAISER VLTTDEIDEHLTAISERD
**16**	100	7	**Proteasome subunit alpha type-2-A** *Arabidopsis thaliana*	25.7	5.53	O23708	**MS/MS (total ion C.I.%: 100)** EPIPVTQLVR NIEIGKIGADK ASAMGKNVSNAK LPSILVDEASVQK KLPSILVDEASVQK (*) LYKEPIPVTQLVR (*) LVQIEHALTAVGSGQTSLGIK
**29**	100	6	**Putative beta4 proteasome subunit** *Nicotiana tabacum*	14.6	6.40	Q93X32	**MS/MS (total ion C.I.%: 97.36)** GELATALR IMILDSHK GELATALRK NVALYQFR (*) LMGASGEAGDR NGIPLTTAAAANFTR

Table S2. Summary of the positive identifications by mass spectrometry of the peak fraction of the sunflower hypocotyls sample eluted from a GSNO-vinyl sulfone resin that showed the highest protein and S-nitrosylation signals. Criteria to consider an identification as positive were: i) agreement between both molecular weigh and isoelectric point inferred from the 2D gel and those reported in literature or predicted from the primary structure and ii) either fragmentation or mass fingerprint yielded a protein score C.I. better than 97% (instead of the less stringent 95% cutoff generally accepted). Peptides labeled as (*) were confirmed by MS/MS

**spot**	**Protein score C.I.%**	**Peptide count**	**Protein** *species*	**MW (kDa)**	**pI**	**ID**	**identification method** matched peptides [(*) Peptides identified by MS/MS]
**3**	99.8	12	**Ferredoxin-nitrite reductase** *Spinacia oleracea*	67.1	6.51	P05314	**Peptide mass fingerprinting**<br>DLGFRGNR<br>NNSSIRCQK<br>RMPEQVLER<br>IADVYGSGELR<br>HHYGRFMMR<br>LQADEMEELAR<br>ERYSPEPPILMK<br>IDSLLNEPLLKER<br>MMWLIDELGMEAFR<br>VTEEVQRLVSVTRPVR<br>LFIEDGISDLATLSMEEVDK<br>AVSPAAETAAVSPSVDAARLEPR
**4**	97.6	9	**Transcriptional adapter ADA2b** *Arabidopsis thaliana*	56.1	6.05	Q9ATB4	**Peptide mass fingerprinting**<br>KAEQNMK<br>EEYPFSPPK<br>ENEEGMNRGK<br>NLLYPNPFEK<br>VDRVYDMLVK<br>EEYPFSPPKVK<br>ENDTPEEHELK<br>RLFSQDMSHVAGK<br>FHSKEEHDELLR
**16**	99.9	11	**Expressed protein** *Oryza sativa*	43.0	5.72	Q2QWE0	**Peptide mass fingerprinting**<br>TMLVEEK<br>LPDCTFGKK<br>TMLSEEKVK<br>LKTMLSEEK<br>LKTMLVEEK<br>TMLVEEKDK<br>LLKTIFVEEK<br>NEHYKTMLAK<br>NMLEEEKDNNK<br>AVEVIQTWSSEVK<br>AEHHKNMLEEEK
**18**	99.9	10	**At2g18860** *Arabidopsis thaliana*	34.0	5.87	Q501A1	**Peptide mass fingerprinting**<br>WQLEEFEK<br>ETLVAQIEATPK<br>IWSSPNFNSLR<br>MVVNSFDLWQK

spot	Protein score C.I.%	Peptide count	**Protein** *species*	MW (kDa)	pI	ID	**identification method** matched peptides [(*) Peptides identified by MS/MS]
							WQLEEFEKAVR<br>QFVTAIENQIHR<br>MMVVNSFDLWQK<br>ESTNSSLVENVMEVSAK (oxid. M12)<br>SRESTNSSLVENVMEVSAK<br>ESTNSSLVENVMEVSAK
**19**	99.9	11	**Pentatricopeptide repeat-containing protein-like protein**<br>*Arabidopsis thaliana*	42.0	8.81	Q9CAY8	**Peptide mass fingerprinting**<br>WGLKYR<br>EKGMVEK<br>LWNEYK<br>AEKLMTR<br>YLDRASR<br>LMTRWGLK<br>MSMTLSLQR<br>VDESLREMK<br>LCSKACIGDK<br>FLADWEGTVK<br>NGTLSTNQTLHGR
**20**	99.6	10	**Hypothetical protein At2g46320**<br>*Arabidopsis thaliana*	39.3	9.37	Q8S8M6	**Peptide mass fingerprinting**<br>AMTMTTR<br>QIEKNTDR<br>MQAFKGTQR<br>SKSIPNENLK<br>GMFSGAGARVGR<br>NTDRAMTMTTR<br>MLWTGLGAQLAR<br>MVEEAQLDPKSK<br>ITGSASVCSDNQYK<br>QTLAEIWRDGGMR
**21**	100	14	**Ferredoxin-nitrite reductase precursor** (fragment)<br>*Spinacia oleracea*	67.1	6.51	P05314	**Peptide mass fingerprinting**<br>SGINPAEK<br>AMLEAFR<br>DGFWVLK<br>LEPRVEER<br>EYLGVHPQK<br>NNSSIRCQK<br>RMPEQVLER<br>HHYGRFMMR<br>IDSLLNEPLLK<br>LQADEMEELAR<br>ERYSPEPPILMK<br>MPEQVLERASSEELVQK<br>LFIEDGISDLATLSMEEVDK<br>MMWLIDELGMEAFRGEVEK
**22**	99.9	4	**Pectinesterase**<br>*Solanum tuberosum*	24.4	8.84	Q43838	**MS/MS (total ion C.I.%: 99.977)**<br>ENVEVTSRK<br>CQLVARKPSK<br>YQKNMVTAQGR<br>IDAYQDTLYAHSQR (*)
**24**	98.7	7	**Catalase 1** (fragment)	34.2	5.9	Q93XK3	**Peptide mass fingerprinting**

spot	Protein score C.I.%	Peptide count	Protein *species*	MW (kDa)	pI	ID	**identification method** matched peptides [(*) Peptides identified by MS/MS]
			Pinus pines				FPDMVHALKPNPK<br>HMDGSGVHTFTLVNK<br>DGMKFPDMVHALKPNPK<br>TWPEDIFPLQPVGRMVLNK<br>GFFEVTHDISHLTCADFLR<br>EGNFDLVGNNFPVFFVRDGMK<br>ILDFFSHHPESLHMFTFLFDDLGIPLNYR
**25**	98	4	**Thioredoxin peroxidase**<br>*Nicotinum tabacum*	30.1	8.2	Q8RVF8	**MS/MS (total ion C.I.%: 99.407)**<br>SVDETLR<br>SYNVLIPDQGIALR (*)<br>SGGLGDLNYPLISDVTK<br>EGVIQHSTINNLGIGRSVDETLR
**26**	99.7	6	**Cytochrome P450**<br>(*fragment*)<br>*Teucrium canadense*	14.6	6.06	Q5EKR9	**Peptide mass fingerprinting**<br>GSNVHVNVWALAR<br>SNANVKIGGYDIPK<br>AQEEMDRVIGYER<br>TSTTMEWAMTELIK<br>FLEEDVDMKGHDFR<br>IGGYDIPKGSNVHVNVWALAR
**29**	99.8	3	**Calmodulin**<br>*Arabidopsis thaliana*	15.6	4.20	P25069	**MS/MS (total ion C.I.%: 99.836)**<br>LTDEEVDEMIK<br>DTDSEEELKEAFR<br>VFDKDQNGFISAAELR (*)
